# Neoadjuvant Therapy or Upfront Surgery for Pancreatic Cancer—To Whom, When, and How?

**DOI:** 10.3390/cancers17152584

**Published:** 2025-08-06

**Authors:** Daria Kwaśniewska, Marta Fudalej, Anna Maria Badowska-Kozakiewicz, Aleksandra Czerw, Andrzej Deptała

**Affiliations:** 1 Department of Oncology, National Medical Institute of the Ministry of the Interior and Administration, 02-507 Warsaw, Poland; daria.kwasniewska@pimmswia.gov.pl (D.K.); marta.fudalej@wum.edu.pl (M.F.); 2 Department of Oncological Propaedeutics, Medical University of Warsaw, 01-445 Warsaw, Poland; anna.badowska-kozakiewicz@wum.edu.pl; 3 Department of Health Economics and Medical Law, Medical University of Warsaw, 02-091 Warsaw, Poland; aleksandra.czerw@wum.edu.pl; 4 Department of Economic and System Analyses, National Institute of Public Health, NIH—National Research Institute, 00-791 Warsaw, Poland

**Keywords:** pancreatic ductal adenocarcinoma cancer, neoadjuvant, adjuvant, resectable, borderline resectable

## Abstract

The management of resectable pancreatic ductal adenocarcinoma (R-PDAC) and borderline resectable pancreatic ductal adenocarcinoma (BR-PDAC) remains a topic of active debate. Although neoadjuvant therapy (NAT) has shown clinical benefits in BR-PDAC, especially in increasing resectability and achieving higher rates of margin-negative (R0) resections, its role in R-PDAC is less clearly defined. Additionally, the role of immunotherapy in PDAC is still being explored, with ongoing trials investigating new combinations to overcome the tumor’s immune-resistant microenvironment. This article provides a comprehensive narrative review of the current evidence comparing NAT with upfront surgery in pancreatic cancer management, focusing on randomized controlled trials and meta-analyses that assess outcomes in R-PDAC and BR-PDAC. The review aims to determine whether NAT offers a significant survival advantage over traditional post-operative strategies and to clarify which clinical scenarios may benefit most from NAT. The literature was identified through a systematic search of PubMed, Scopus, and Google Scholar databases up to March 2025. Article selection adhered to the PRISMA guidelines. Our review of existing evidence supports NAT as the standard of care for BR-PDAC. Meanwhile, management of R-PDAC should be tailored individually, guided by risk stratification that considers both clinical parameters and molecular features. Immunotherapy and targeted therapies are still in early research phases, and their further integration as NAT remains controversial.

## Simple Summary

Pancreatic ductal adenocarcinoma (PDAC) remains one of the most lethal malignancies, with a five-year survival rate below 10%. Even in patients diagnosed at an early stage, when the tumor is deemed technically resectable, long-term outcomes remain unsatisfactory due to high rates of local recurrence and distance metastases. This article provides a comprehensive narrative review of the current evidence comparing neoadjuvant therapy (NAT) with upfront surgery in pancreatic cancer management. The literature was identified through a systematic search of PubMed, Scopus, and Google Scholar databases up to March 2025. Our review of existing evidence supports NAT as the standard of care for borderline resectable PDAC. Meanwhile, management of resectable PDAC should be tailored individually, guided by risk stratification that considers both clinical parameters and molecular features. Immunotherapy and targeted therapies are still in early research phases, and their further integration as NAT remains controversial.

## 1. Introduction

Pancreatic carcinoma (PC) remains one of the most aggressive and lethal solid malignancies, exhibiting minimal improvements in survival rates despite advancements in surgical techniques and systemic therapies. The predominant histological type is pancreatic ductal adenocarcinoma (PDAC), which will be the primary focus of this article. The global incidence of PDAC is increasing, currently ranking as the third leading cause of cancer-related mortality in Europe and the seventh in the United States [1,2]. With projections indicating that PDAC will ascend to the second leading cause of cancer-related deaths by the year 2030, advancing research in this field is more urgent than ever. Unfortunately, most patients are diagnosed at an advanced stage, which significantly worsens the prognosis. Only approximately 10–20% of PDAC cases are diagnosed at an early stage, wherein treatments such as radiotherapy, chemotherapy, or surgical interventions appear to enhance patient outcomes [3,4]. Earlier detection greatly improves outcomes. Patients diagnosed at stage I have a 5-year survival rate of about 30–60%, compared to less than 5% for those with metastatic disease [3]. Early-stage tumors are more likely to respond favorably to neoadjuvant or adjuvant therapies, increasing the chances of complete resection and long-term survival [5]. Metastases are a key factor in determining prognosis in PC; understanding the mechanisms behind metastatic progression and developing effective screening and treatments are urgent priorities in managing this cancer. Some preclinical studies investigate biomarkers for early cancer diagnosis, such as the study by Ren et al. [6] (2025), where elevated levels of serum exosomal hsa-let-7f-5p in metastatic PC indicated its potential as a non-invasive biomarker for distinguishing metastatic from non-metastatic disease.

PDAC frequently recurs even after radical resection, which highlights the critical need for peri-operative treatments aimed at enhancing patient outcomes. The 5-year survival rate without peri-operative treatment is approximately 10%, while the integration of adjuvant therapy has raised these rates to between 20% and 40% over the past two decades [7,8].

Therapeutic strategies are primarily determined by the anatomical eligibility of the tumor for surgical intervention, which involves assessing the extent of vascular involvement [9] as well as biological and conditional aspects [10]. Tumor encasement involving the vasculature surrounding the pancreas presents considerable technical challenges for surgical resection and is linked to a poorer prognosis. Consequently, PDAC is categorized into resectable (R-PDAC), borderline (BR-PDAC), and locally advanced (LA-PDAC) classifications [11]. NCCN criteria for PDAC staging are presented in Table 1 [12].

Regardless of the initial classification, surgical resection remains the primary goal of curative treatment. Latenstein et al. presented that the 1-year conditional survival increased progressively from 55% at 1 year to 74% at 3 years, and 86% at 5 years following surgical resection. Similarly, median overall survival (mOS) improved from 15 months to 40 months and 64 months at the respective post-operative intervals [13].

However, the optimal sequencing of surgery, systemic therapy, or radiotherapy remains a subject of ongoing clinical investigation. This article presents a comprehensive narrative review of the current evidence comparing neoadjuvant therapy (NAT) with upfront surgery in the management of pancreatic cancer, with a particular focus on randomized controlled trials (RCTs) and meta-analyses that evaluate treatment outcomes in R-PDAC and BR-PDAC. The review aims to determine whether NAT provides a significant survival benefit over conventional post-operative strategies and to clarify the clinical scenarios where NAT may be most advantageous. Simultaneously, the growing role of immunotherapy in treating various solid tumors raises the question of whether it may also play a role in managing early-stage pancreatic cancer, either alone or in combination with existing therapies. The primary objective of this review is to systematically analyze data from completed clinical trials investigating adjuvant, neoadjuvant, and peri-operative strategies in patients with R-PDAC and BR-PDAC. By analyzing the outcomes of these trials, we seek to identify the most effective therapeutic approaches and provide guidance for future clinical practice and research directions.

The literature was identified through a systematic search of the PubMed, Scopus, and Google Scholar databases up to March 2025. Article selection followed the Preferred Reporting Items for Systematic Reviews and Meta-Analyses (PRISMA) guidelines. We included randomized clinical trials and meta-analyses that focused on adjuvant and neoadjuvant strategies in PDAC. The search strategy incorporated multiple combinations of terms such as “neoadjuvant,” “adjuvant,” “resectable,” “borderline resectable,” “pancreatic cancer,” and “FOLFIRINOX,” and was limited to studies published in English. This manuscript is based on a review of the existing literature and did not involve the collection of original data, human participants, or animal subjects. Accordingly, ethical approval was not required, and the study was deemed exempt from institutional review in accordance with local and international guidelines.

## 2. Evaluation of the Role of Neoadjuvant, Adjuvant, and Surgical Treatment of R-PDAC and BR-PDAC over the Years in Light of Clinical Studies

Over the past decade, the role of adjuvant therapy in the treatment of PDAC has been well established, whereas NAT approaches, particularly in R-PDAC, remain the subject of ongoing clinical trials. Current efforts are focused on optimizing neoadjuvant strategies to increase the proportion of resectable cases, improve the likelihood of R0 resections, OS, and facilitate more precise selection of patients who may benefit from surgical intervention. Given the significant therapeutic challenges and persistent poor prognosis associated with PDAC, an in-depth and comprehensive analysis of evolving clinical evidence is essential to support evidence-based decision-making and guide individualized treatment planning.

### 2.1. Historical Standard of Care in PDAC Treatment

The first clear therapeutic benefit of adjuvant treatment was established after the outcomes of the ESPAC-1 trial in 2004 [14]. The study demonstrated a significant survival advantage with adjuvant 5-fluorouracil plus leucovorin (5-FU+LV) therapy compared to resection alone, thereby establishing 5-FU+LV as the standard adjuvant treatment for resectable PDAC. Following the ESPAC-1 trial, the CONKO-001 trial randomized patients’ post-resection to receive either adjuvant gemcitabine (GEM) or observation. The study reported a significant improvement in recurrence-free survival (RFS) (13.4 months vs. 6.9 months, *p* = 0.001) in the GEM group. Although initial analysis did not show a statistically significant improvement in OS, long-term follow-up eventually revealed a significant OS benefit as well (22.8 months vs. 20.2 months, *p* = 0.01) [8]. 

Around the same period, the JSAP-02 trial in Japan evaluated adjuvant GEM therapy in 118 patients following resection for PDAC. Similar to CONKO-001, JSAP-02 showed a significant improvement in RFS with GEM [15]. These findings collectively led to the recognition of GEM as an alternative standard adjuvant therapy alongside 5-FU+LV. In contrast, the ESPAC-3 trial, conducted by the European Study Group for Pancreatic Cancer compared GEM with 5-FU+LV in 1088 patients receiving adjuvant therapy. The study found no significant difference in OS between the two treatment arms (23.6 months vs. 23.0 months; HR 0.94; 95% CI, 0.81–1.08; *p* = 0.39). Due to significantly lower incidence of serious adverse events, GEM demonstrated a safer profile and was subsequently adopted as the standard adjuvant chemotherapy in clinical practice [16]. Randomized controlled trials comparing adjuvant chemotherapy with observation for PDAC are depicted in Table 2.

### 2.2. Actual Standard of Care and Challenges in PDAC Treatment

Currently, the standard of care for patients with R-PDAC involves surgical resection followed by adjuvant therapy with mFOLFIRINOX, as demonstrated by the PRODIGE 24 randomized trial, which showed a significant survival advantage. Patients were randomized to receive adjuvant therapy with FOLFIRINOX or gemcitabine. Median disease-free survival (DFS) was 21.4 months (95% CI, 17.5–26.7) vs. 12.8 months (95% CI, 1.6–15.2) (HR 0.66; 95% CI, 0.54–0.82; *p* = 0.001), and median OS (mOS) was 53.5 months (95% CI, 43.5–58.4) vs. 35.5 months (95% CI, 30.1–40.3) (HR 0.68; 95% CI, 0.54–0.85; *p* = 0.001) [17]. For patients who are not eligible to receive mFOLFIRINOX due to unfit conditions or comorbidities, adjuvant therapy with GEM and capecitabine, as established in the ESPAC-4 trial, remains the standard of care. The trial demonstrated an improvement in mOS at 28.0 months vs. 25.5 months, respectively (Table 3) [18].

Considering that only about 50% of patients receive adjuvant therapy, often due to post-operative complications or delayed recovery, a shift toward NAT appears justified. Neoadjuvant treatment is expected to enable the earlier administration of systemic therapy, potentially increasing the proportion of patients receiving systemic treatment and subsequently improving OS. Additionally, this approach may help avoid ineffective surgical interventions in patients with hidden metastases or rapidly progressing diseases. However, a significant portion of patients who start NAT ultimately do not undergo surgical resection [19]. The main reasons for patients not undergoing surgery after neoadjuvant treatment include disease progression, insufficient general condition to undergo surgery, or patient preference [20].

### 2.3. Clinical Trial Results—Looking for the Best Therapeutic Option

Despite significant efforts, outcomes in the treatment of PDAC remain suboptimal, even in patients at early stages undergoing resection followed by adjuvant therapy. Very high recurrence rates and low OS necessitate the search for more effective treatment strategies [21]. In this context, administering NAT before surgery has gained recognition as a promising strategy, especially for BR-PDAC, and is increasingly being considered even in R-PDAC [22]. This section presents the results of recent studies comparing NAT, upfront surgery, and peri-operative approaches to assess their impact on resectability and survival outcomes.

One of the first randomized clinical trials for NAT in PDAC was conducted by Golcher et al. [23] in 2015. Although the trial was terminated due to slow recruitment, and the results were not significant, it demonstrated that NAT with chemoradiotherapy (CRT) is safe, exhibiting acceptable toxicity as well as peri-operative morbidity and mortality rates. Casadei et al. [24] (2015) also demonstrated that NAT was feasible and safe; however, due to poor accrual, the study was terminated early. In 2018, Jang et al. [25] conducted a prospective, randomized phase II/III trial involving 110 patients with BR-PDAC. Participants were randomly allocated to receive neoadjuvant CRT with GEM followed by surgery, or to undergo surgery followed by CRT. The results showed a benefit in 2-year survival (40.7% vs. 26.1%) and OS (21 months vs. 12 months, *p* = 0.028) for neoadjuvant gemcitabine-based CRT over upfront surgery in the Intention-to-Treat (ITT) analysis. The study also showed a significantly higher R0 resection rate following neoadjuvant chemotherapy (51.8% vs. 26.1%, *p* = 0.004). Similarly, the Japanese prep-2/JSAP-05 trial (2019) enrolled 364 patients, who were randomized to receive either NAT chemotherapy with GEM and oral fluoropyrimidine (S-1) followed by surgery and adjuvant S-1, or upfront surgery followed by adjuvant S-1. OS was significantly improved in the NAT group (HR 0.72; 95% CI, 0.55–0.94; *p*  =  0.015) [26]. In turn, in the Dutch PREOPANC trial (2022) randomized 246 patients with R-PDAC and BR-PDAC to receive either neoadjuvant CRT with GEM followed by surgery and adjuvant GEM, or upfront surgery with subsequent GEM. The results showed improvement in OS in the CRT group compared to the upfront surgery group (15.7 months vs. 14.3 months; HR 0.73; 95% CI, 0.56–0.96, *p* = 0.025). Furthermore, patients in the NAT arm achieved higher 5-year OS rates (20.5% vs. 6.5%), and R0 resection rates (71% vs. 49%, *p* = 0.001) [27]. A more recent study, PREOPANC-2 (2023), a phase III trial, analyzed FOLFIRINOX treatment regimen as the NAT (8 cycles) without adjuvant therapy versus gemcitabine with radiotherapy during the second cycle, followed by surgery and adjuvant gemcitabine. Nevertheless, in this study, NAT with FOLFIRINOX failed to improve OS (21.9 months vs. 21.3 months, respectively; HR 0.87; 95% CI 0.68–1.12, *p* = 0.28) [28,29].

The NORPACT-1 trial (2024), one of the first randomized controlled trials, compared the role of neoadjuvant FOLFIRINOX with upfront surgery in patients with R-PDAC. The results did not show a significant survival benefit from NAT compared to upfront surgery. The mOS in the neoadjuvant group was 25.1 months, while in the upfront surgery group, it was 38.5 months (HR 1.52; CI 95% 1.00–2.31, *p* = 0.050). Additionally, the proportion of patients alive after 18 months was 60% in the neoadjuvant group compared to 73% in the upfront surgery group (*p* = 0.10) [30]. In the SWOG S1505 trial (2021), patients with R-PDAC were randomized to receive one of the pre-operative treatment regimens: mFOLFIRINOX (arm 1) or GEM/nab-paclitaxel (arm 2). The estimated 2-year OS was 47% (95% CI, 31–61%; *p* = 0.15) for mFOLFIRINOX and 48% (95% CI, 31–63%; *p* = 0.14) for GEM/nab-paclitaxel group. The median OS was 23.2 months (95% CI, 17.6–45.9 months) and 23.6 months (95% CI, 17.8–31.7 months), in arms 1 and 2, respectively [31]. The NEONAX trial (2023), a phase II prospective randomized study, evaluated peri-operative chemotherapy versus adjuvant therapy with gemcitabine and nab-paclitaxel in patients with R-PDAC. The primary endpoint of the study was the 18-month DFS rate in the modified ITT population, which was not achieved in either group. DFS was 33.3% (95% CI: 18.5–48.1%) in the neoadjuvant group (arm A) and 41.4% (95% CI: 20.7–62.0%) in the adjuvant group (arm B). The mOS, assessed as a secondary endpoint in the ITT population, numerically favored the neoadjuvant treatment group (arm A) with a median OS of 25.5 months (95% CI: 19.7–29.7) compared to 16.7 months in arm B (95% CI: 11.6–22.2). It is worth noting the significant difference in chemotherapy exposure between the groups: 90% of patients in arm A completed pre-operative chemotherapy, while only 58% in arm B started adjuvant therapy. Additionally, 88% of patients in the neoadjuvant arm achieved R0 resection, compared to 67% in the adjuvant arm. These findings support the potential of neoadjuvant or peri-operative therapy as a treatment strategy in R-PDAC; however, the failure to meet the DFS target highlights the need for further research to refine treatment strategies and optimize patient outcomes, especially as the optimal regimen has yet to be clearly defined [32]. The PANACHE01-PRODIGE48 study (2025) further confirmed that NAT chemotherapy with FOLFIRINOX is both feasible and effective in patients with R-PDAC. Patients were randomized into two arms: (1) FOLFIRINOX, (2) leucovorin, fluorouracil, and oxaliplatin (FOLFOX), and upfront surgery as control. Within 12 months post-randomization, 84.3% (90% CI, 75.3–90.9) and 71.4% (90% CI, 59.0–81.8) of the patients were alive in arm 1 and arm 2, respectively. Arm 2 was stopped after the interim analysis for lack of efficacy. One-year EFS rates were 51.4% (95% CI, 41.0–64.3), 43.1% (95% CI, 31.3–59.5), and 38.7% (95% CI, 24.1–62.0) in arm 1, arm 2, and control arm, respectively [33]. In an earlier study—ALLIANCE A021501 (2022), 126 patients were enrolled to compare treatment with neoadjuvant mFOLFIRINOX with or without hypofractionated radiation therapy in BR-PDAC. The study reported the mOS of 29.8 months (95% CI, 21.1–36.6) in arm 1, compared to 17.1 months (95% CI, 12.8–24.4) in arm 2, respectively. These findings indicated that treatment with neoadjuvant mFOLFIRINOX alone was associated with improved survival outcomes in patients with BR-PDAC compared to mFOLFIRINOX followed by hypofractionated radiotherapy [34]. At the 2025 American Society of Clinical Oncology (ASCO) Annual Meeting, investigators presented the interim results from the phase III CASSANDRA trial, which compared neoadjuvant mFOLFIRINOX with PAXG (cisplatin, nab-paclitaxel, capecitabine, and gemcitabine) in patients with R-PDAC and BR-PDAC. PAXG regimen was associated with a significantly improved 3-year EFS rate of 31%, compared with 13% in the mFOLFIRINOX group (HR = 0.64; 95% CI: 0.48–0.86; *p* = 003). The median EFS was 16.0 months with PAXG versus 10.2 months with mFOLFIRINOX [35]. Selected randomized controlled trials comparing NAT with upfront surgery are presented in Table 4.

### 2.4. Meta-Analyses of Clinical Trials

As there is no consensus on the integration of NAT into the standard of care for patients with PC, various authors have conducted meta-analyses on this topic. Comparing different studies in a meta-analysis provides a systematic and quantitative summary, reducing the influence of outlier results. Consequently, meta-analyses may also yield clinical guidelines [36]. A summary of the key findings from the meta-analysis focused on NAT in PC is presented below.

Versteijne et al. [37] (2018) conducted a meta-analysis of 38 studies (3484 patients) with R-PDAC and BR-PDAC comparing outcomes of upfront surgery versus NAT (chemotherapy—usually including gemcitabine or radiotherapy as a part of combination treatment). The mOS was 18.8 months in the NAT arm vs. 14.8 months in the upfront surgery arm in the ITT analysis. Moreover, the difference was larger among patients who underwent surgery (26.1 months vs. 15 months). Although the overall resection rate was lower in the NAT arm at 66.0% compared to the upfront surgery arm at 81.3% (*p* = 0.001), the R0 rate was significantly higher, reaching 86.8% (95% Cl, 84.6–88.7) versus 66.9% (95% Cl, 64.2–69.6). Reported by ITT, the R0 rates were 58% compared to 54.9% (*p* = 0.088), respectively. These findings suggest that NAT confers a survival benefit despite a reduced likelihood of undergoing surgical resection.

Aliseda et al. [38] analyzed patients with R-PDAC. The meta-analysis of five prospective randomized clinical trials (Golcher et al. [23], PACT-15, PREP-02/JSAP-05, PREOPANC, NEONAX) included 625 patients, of whom 272 underwent upfront surgery and 353 received NAT. Among the NAT group, 288 patients received chemotherapy alone, and 65 underwent CRT. The most commonly administered chemotherapy regimen was FOLFIRINOX (n = 147), followed by FOLFOX (n = 50), and various gemcitabine-based protocols (n = 156). Among the 625 patients, the mOS of patients in the NAT and upfront surgery groups was 25.8 months (95% CI, 21.4–29.9) and 22.1 months (95% CI, 18.3–26.5), respectively. The study indicated no significant difference in the hazard of death between the NAT and upfront surgery groups (HR: 0.88, 95% Cl 0.72–1.08, *p* = 0.233). Similarly, the non-parametric restricted mean survival time model demonstrated a modest difference (+2.41 months, 95% Cl 1.22 to 6.04, *p* = 0.194).

Similar results were presented by Uson Junior et al. [39]. They conducted a meta-analysis encompassing six randomized clinical trials, which included a combined group of 805 patients with R-PDAC. Among the studies analyzed were PACT-15, PREP-02/JSAP-05, PREOPANC, and NEONAX trials. The pooled results indicated that NAT did not confer a statistically significant advantage in DFS (HR: 0.71; 95% CI: 0.46–1.09, *p* = 0.115) or OS (HR: 0.76; 95% CI: 0.52–1.11, *p* = 0.150), with no evidence of significant heterogeneity among the included studies. Notably, the primary benefit of the NAT approach was an improvement in R0 resection rates. Overall, neoadjuvant chemotherapy or CRT increased the likelihood of R0 resection by approximately 20% (HR: 1.20; 95% CI: 1.04–1.37). It is essential to note that the majority of trials included in their meta-analysis used gemcitabine-based regimens.

The findings discussed above suggest that NAT does not lead to a significant survival benefit over upfront surgery for patients with R-PDAC. The most recent meta-analysis presented by Dickerson et al. [40] (2025) included nine randomized controlled trials comprising a total of 1194 patients with R-PDAC and BR-PDAC. The therapeutic modalities assessed NAT in four trials, CRT in four, and a combination of both in one trial. The analysis revealed that NAT was associated with a significant improvement in OS (HR: 0.73; 95% CI, 0.55–0.98; *p* = 0.001) and DFS (HR: 0.80; 95% CI, 0.65–0.99; *p* = 0.041). Subgroup analysis demonstrated a survival benefit for patients with BR-PDAC (HR: 0.60; 95% CI 0.38–0.96), whereas no significant benefit was observed in the R-PDAC subgroup (HR 0.90; 95% CI, 0.63–1.28). Regarding surgical outcomes, the overall resection rate was slightly lower in the NAT group compared to the upfront surgery group (72.6% vs. 80.6%; 95% CI, 0.89–0.99; *p* = 0.020). However, NAT significantly improved the rate of R0 resections (43.8% vs. 23.0%; 95% CI, 1.16–1.57; *p* = 0.002) and nodal negativity (N0) (30.9% vs. 15.0%; 95% CI, 1.50–2.74; *p* = 0.001).

In turn, another meta-analysis by Chan et al. [41] (2025) demonstrated that NAT in patients with R-PDAC significantly improved DFS compared to upfront surgery and was associated with a higher resection rate. The analyses included eight randomized controlled trials enrolling 982 patients. Patients treated with NAT showed a significantly better mDFS compared to those undergoing upfront surgery (HR: 0.66; 95% CI,0.47–0.92, *p* = 0.01) with significantly improved R0 resection rate (Relative Risk RR = 1.20; *p* = 0.04). However, there was not observed significant difference in OS between both groups (HR: 0.01; *p* = 0.21). These findings highlight the potential benefits of NAT in surgical and disease control, though further studies are needed to confirm its impact on OS.

In the 2020 meta-analysis, Cloyd et al. [42] evaluated six randomized trials comparing NAT with upfront surgery in patients with R-PDAC and BR-PDAC. The findings revealed a significant OS advantage in favor of NAT based on an ITT analysis (HR: 0.73; 95% CI, 0.61–0.86). The survival benefit was observed across all subgroups: in the R-PDAC (HR: 73; 95% CI, 0.59–0.91), BR-PDAC (HR: 0.77; 95% CI, 0.28–0.93), as well across different treatment strategies: CRT (HR: 0.77;95% CI, 0.61–0.98) and chemotherapy alone (HR: 0.68; 95% CI, 0.54–0.87). Additionally, patients receiving NAT were significantly more likely to achieve an R0 resection. A review of the described meta-analysis is depicted in Table 5.

While some studies suggest the potential benefits of NAT in improving survival outcomes and surgical efficacy, others highlight the need for more research to confirm these findings. It is hoped that the results of these ongoing studies will provide valuable insights into the most effective treatment strategies for patients (Table 6).

## 3. Current Recommendations for Neoadjuvant Therapy and Upfront Surgery in Pancreatic Cancer

NAT was initially introduced to provide potential advantages in tumor downstaging before surgical resection; however, it is now predominantly used to identify patients whose tumor biology indicates a greater benefit from NAT compared to upfront surgery. This approach has additionally resulted in an increase in margin-free resection (R0), which is correlated with enhanced treatment outcomes. Finally, all of these factors contribute to a reduction in the risk of recurrence following curative surgery. The determination of resectability in patients with PDAC should be established through a multidisciplinary consensus at high-volume, specialized centers, following the acquisition of high-quality, dedicated pancreatic imaging and comprehensive clinical staging. The timing and modality of imaging should be tailored to the clinical context and conducted either before surgery, within four weeks of the planned operation, or after completing NAT to ensure an accurate assessment of tumor extent and resectability. The preferred imaging modality is multidetector computed tomography (MDCT) angiography, which uses a pancreas-specific protocol. This procedure involves the acquisition of thin (ideally sub-millimeter) axial slices during both the pancreatic and portal venous phases of contrast enhancement. It is highly recommended to use multiplanar reconstructions to facilitate detailed visualization of the tumor’s anatomical relationship with the surrounding vasculature, including the superior mesenteric artery (SMA), celiac axis, and portal venous system, thereby improving the detection of subcentimeter metastatic lesions. Magnetic resonance imaging (MRI) is primarily used for characterizing indeterminate hepatic lesions identified on computed tomography (CT), especially in scenarios where pancreatic tumors are not distinctly visible on CT or in instances where contrast-enhanced CT is contraindicated [43].

According to the National Comprehensive Cancer Network (NCCN) guidelines, patients with R-PDAC should be considered for upfront surgery in the absence of arterial tumor contact with the celiac axis, SMA, or common hepatic artery (CHA), and with no venous involvement or ≤180° contact with the superior mesenteric vein (SMV) or portal vein (PV), provided there is no contour irregularity. Adjuvant chemotherapy with modified FOLFIRINOX (mFOLFIRINOX) is regarded as the preferred treatment option, whereas the combination of gemcitabine and capecitabine is considered a viable alternative option [44]. This approach is supported by the PRODIGE 24 trial, which demonstrated improved OS with adjuvant FOLFIRINOX compared to gemcitabine alone [17]. In cases where high-risk features are present, such as markedly elevated CA 19–9 levels, large primary tumors, bulky regional lymph nodes, significant weight loss, or severe pain, neoadjuvant chemotherapy with mFOLFIRINOX should be considered. For patients with BR-PDAC, the NCCN guidelines strongly recommend NAT as the first-line approach, even if the tumor appears technically resectable [44]. This recommendation is supported by data from the PREOPANC and Alliance A021501 trials [40].

In contrast, the National Institute for Health and Care Excellence (NICE) in the United Kingdom recommends that NAT for both R-PDAC and BR-PDAC should be considered only within the framework of clinical trials. Surgery followed by adjuvant chemotherapy remains the standard of care for both groups. However, it is worth noting that the NICE guidelines have not been updated since 2018 [45].

The European Society for Medical Oncology (ESMO) states that surgical resection remains the standard treatment for patients with R-PDAC, followed by adjuvant chemotherapy to improve survival outcomes. Recommended regimens include FOLFIRINOX or gemcitabine-based combinations, depending on the patient’s performance status and tolerance. For BR-PDAC, ESMO strongly endorses NAT to enhance resectability and survival rates. Recommended protocols encompass FOLFIRINOX or gemcitabine/nab-paclitaxel, with or without CRT, tailored to individual patient characteristics. This approach aims to downstage the tumor, potentially allowing for surgical resection. Following neoadjuvant treatment, patients should be reevaluated for surgical resectability, and resection should be pursued if deemed feasible [46].

These recommendations are based on the best available scientific evidence and expert consensus, emphasizing the importance of a multidisciplinary approach in managing PC. Engaging in shared decision-making with patients is crucial for tailoring treatment plans to individual patient characteristics and preferences. Most of the presented studies primarily focus on outcomes such as OS, DFS, and resection efficacy. Unfortunately, aspects related to quality of life, functional status, and treatment tolerability are often overlooked in these analyses. Meanwhile, patients with pancreatic cancer frequently present with frailty and multiple comorbidities, making these factors particularly important for an accurate assessment of the true value of therapeutic interventions. The studies of Seufferlin et al. [32] (2023) and Sohal et al. [31] (2021) emphasize that including these factors is crucial for creating personal treatment plans that not only prolong survival but also preserve a good quality of life.

## 4. Neoadjuvant Immunotherapy

Tumor immunotherapy has significantly transformed the treatment landscape for various solid tumors; however, its efficacy in PDAC is still constrained. The observed immunological resistance of PDAC is ascribed to a multifactorial interplay, which includes a low tumor mutational burden and a highly immunosuppressive tumor microenvironment (TME). This TME is characterized by dense desmoplastic stroma, hypoxia, inadequate infiltration of cytotoxic T lymphocytes, and the presence of immunosuppressive immune cells [47]. The inflammatory process serves as a critical mediator in the pathogenesis of PDAC, contributing to tumor initiation, progression, and immune evasion. Consequently, PDAC remains one of the most immune-resistant malignancies, and the clinical benefits of immunotherapy, particularly immune checkpoint inhibitors (ICIs), are subject to rigorous investigation. Recent biological insights into the complexities of this microenvironment have revealed new opportunities for integrating therapeutic strategies to overcome resistance. One promising approach entails the neoadjuvant administration of ICIs, which may theoretically provide greater therapeutic benefit by priming an adaptive immune response against tumor-associated antigens while the primary tumor remains present [48,49]. In cancers characterized by high immunogenicity, neoadjuvant ICI therapy has been associated with significant pathological responses, indicating that early modulation of the immune system may enhance anti-tumor activity. In this context, recent research has commenced examinations into the potential of integrating ICIs into neoadjuvant chemotherapy regimens to promote local tumor downstaging in PC. These combination strategies aim to exploit possible synergistic effects between chemotherapy-induced immunogenic cell death and immune activation, thereby improving treatment efficacy in this otherwise highly refractory disease [50].

Du et al. [51] (2023) presented in their phase II study that the combination of NAT PD-1 blockade (tislelizumab) with chemotherapy (gemcitabine and nab-paclitaxel), and stereotactic radiotherapy with simultaneous integrated boost (SBRT-SIB), is a promising pre-operative treatment strategy for patients with BR-PDAC or locally advanced pancreatic cancer (LAPC). The combined treatment achieved an objective response rate (ORR) of 60%, an R0 resection rate of 90%, a 12-month PFS of 64%, and OS of 72%. These results suggest that integrating immunotherapy with CRT in NAT may improve surgical outcomes and long-term prognosis. Moreover, analysis of circulating tumor DNA (ctDNA) suggested that patients with a greater than 50% decrease in maximum somatic variant allele frequency (maxVAF) between baseline and the first assessment had better survival outcomes, higher response rates, and higher rates of surgical resection. Another very promising study was shown by Heumann et al. [52] (2023). Their study assessed the safety and efficacy of the GVAX vaccine in combination with low-dose cyclophosphamide alone (arm A), with the addition of the anti-PD-1 antibody nivolumab (arm B), and with both nivolumab and the anti-CD137 agonist antibody urelumab (arm C) in patients with R-PDAC. The primary objective was to promote enhanced infiltration of cytotoxic CD8+ T cell infiltration into the TME. The mDFS were 13.90/14.98/33.51 months and median OS 23.59/27.01/35.55 months, respectively, for arms A/B/C. Although the difference observed in arm C was impressive (HR for DFS 0.55 vs. A; HR for OS 0.59 vs. A), it did not reach statistical significance (*p* = 0.17 and *p* = 0.38) due to the small group size. Furthermore, the results indicated that the triplet combination (GVAX + PD-1 + CD137) achieved a significant increase in clonal expansion and activation within intratumoral CD8+CD137+ T cells.

Neoadjuvant administration of selicrelumab, an agonist CD40 monoclonal antibody, was evaluated in a phase I/II trial in patients with BR-PDAC [53]. The main goal of the study was to assess the impact of CD40 activation on TME, both as monotherapy and in combination with chemotherapy (gemcitabine and nab-paclitaxel), before surgery, followed by adjuvant treatment with chemotherapy and CD40 antibody. The toxicity profile was well tolerated, and the OS was 23.4 months (95% CI, 18.8–28.8). The selicrelumab changed the TME, showing that 82% of treated tumors were T cell-enriched compared to 37% (*p* = 0.004) in untreated controls and 23% (*p* = 0.012) in chemo/radiotherapy-treated group. There was a significant reduction in tumor fibrosis, suppression of M2-like tumor-associated macrophages, and increased maturation of intratumoral dendritic cells. Importantly, CD8^+^ T cells displayed increased activation and clonal expansion both intratumorally and systemically. These findings support further investigation of CD40 agonists as part of the NAT strategy, particularly in combination with chemotherapy or checkpoint blockade, aiming to transform the immunologically “cold” pancreatic tumor into a more immunologically active disease. A summary of the discussed trials is presented in Table 7.

The CISPD-4 clinical trial evaluated neoadjuvant treatment with FOLFIRINOX, either with or without a programmed cell death protein 1 (PD-1) inhibitor, in patients with BR-PDAC or LAPC. Radiological assessments prior to surgery indicated a partial response in 36% of patients receiving the PD-1 inhibitor, compared to 13% in the chemotherapy-only cohort. Approximately 50% of patients in each group underwent surgical resection. Notably, among those with LA disease, the conversion rate to resectability was higher in the PD-1 group (48% vs. 37%). Furthermore, R0 resections were achieved more frequently in the PD-1 cohort (87% vs. 70%). Long-term survival outcomes have yet to be reported (NCT03983057) [54].

Finally, it needs to be pointed out that the above studies are small phase I/II trials with limited sample sizes, which reduces their statistical power. Some trials, such as CISPD-4, have not yet reported long-term survival data, making it difficult to assess sustained benefit. In the Heumann trial, although improved DFS and OS were observed, statistical significance was not reached due to the small cohort size. Therefore, larger, randomized phase III trials with standardized endpoints, longer follow-up, and detailed toxicity assessments are essential. Future studies should also focus on identifying predictive biomarkers to guide patient selection. Until such data are available, the clinical use of neoadjuvant immunotherapy in PDAC should be considered experimental.

Some ongoing clinical trials of immunotherapy for PDAC are presented in Table 8.

## 5. Neoadjuvant Targeted Therapies

PDAC is a heterogeneous disease characterized by four common genetic alterations: KRAS oncogenic activation and inactivation of the tumor suppressor genes CDKN2A, SMAD4, and TP53. Each of these mutations has specific consequences at the molecular level and affects the functioning of cells, including uncontrolled cell division [55]. The presence of specific mutations influences the planning of therapeutic procedures and patient prognosis. Moreover, PDAC is a tumor with extensive activation of multiple molecular pathways that lead to growth and metastasis. These signaling pathways contribute to uncontrolled cellular proliferation. There are several main pathways associated with specific alterations that offer potential therapeutic targets. The overactivation of the RAS-MAPK pathway is caused by activating mutations in the KRAS gene, which occurs in more than 90% of cases and drive cell growth and proliferation [56]. The second pathway is the PI3K-AKT-mTOR pathway, which is co-activated with the MAPK pathway, promoting cell survival, metabolism, and resistance to apoptosis [57]. The epidermal growth factor receptor (EGFR) pathway intersects with the insulin growth factor (IGF-1) pathway, leading to the proliferation and migration of cancer cells [58]. Additionally, a subset of PDAC is characterized by the activation of angiogenic pathways involving vascular endothelial growth factor (VEGF) secretion and its receptors. This increase in neovascularization in the tumor microenvironment leads to tumor expansion. Finally, there is also a subgroup of patients with BRCA1/2 mutations who suffer from DNA repair deficiency, which is a potential target for the development of molecular therapy [59,60]. Some ongoing clinical trials for PDAC are presented in Table 9.

## 6. Discussion

The treatment landscape for resectable and borderline resectable disease is constantly evolving. While significant progress has been made, numerous challenges remain in establishing standardized treatment algorithms. Across clinical trials and meta-analyses, substantial variability in patient selection, NAT regimens, and criteria for tumor resectability complicates direct comparisons and limits definitive conclusions. These inconsistencies highlight the need for careful interpretation of trial outcomes and emphasize the importance of individualized treatment planning. Differences in outcomes between the PREOPANC, NORPACT-1, and NEONAX trials may be attributed to variations in patient selection, treatment regimens, and trial design. PREOPANC included both R-PDAC and BR-PDAC patients and involved CRT, which could contribute to improved R0 resection rates and OS by enhancing local control and enabling biological selection. In contrast, NORPACT-1 focused solely on clearly R-PDAC and used neoadjuvant FOLFIRINOX without radiotherapy, with a substantial proportion of patients failing to reach surgery, thereby diluting the potential benefit of NAT. The NEONAX study, with NAT using gemcitabine plus nab-paclitaxel, enrolled patients with R-PDAC and also showed no survival advantage. These findings suggest that the benefit of NAT is more evident in BR-PDAC, while its role in R-PDAC remains inconclusive and regimen-dependent.

In patients with R-PDAC, NAT has been shown to improve surgical outcomes, especially by increasing R0 resection rates. Trials like NEONAX and meta-analyses by Aliseda and Chan reported significant improvements in margin-negative resection in the NAT groups compared to upfront surgery. However, these benefits did not consistently lead to better OS. In fact, the NORPACT-1 trial showed worse OS in the NAT group, likely due to patient dropout, treatment delays, and disease progression during NAT. Several meta-analyses (e.g., Uson, Dickerson) reported no notable OS advantage in R-PDAC, highlighting variability based on chemotherapy regimen, patient selection, and study design. Only Cloyd et al. [42] observed a meaningful OS benefit in R-PDAC, suggesting that the benefit may exist in specific subgroups. Therefore, in R-PDAC, the role of NAT remains controversial: it provides technical advantages but lacks consistent evidence of survival benefit. There are also concerns about delayed surgery and the potential loss of operability in patients whose disease progresses.

In contrast, the evidence for BR-PDAC strongly supports using NAT. Multiple randomized trials, including Jang, PREOPANC, and meta-analyses by Dickerson and Cloyd, have shown that NAT significantly improves both R0 resection rates and OS in this patient group. Jang reported a doubling of mOS (21 vs. 12 months) and a dramatic increase in R0 rates (52% vs. 26%) with NAT. The rationale is clear: BR-PDAC is frequently associated with vascular involvement and limited resectability upfront. NAT downstages tumors, improves margin clearance, and allows for biological selection by excluding early progressors. Importantly, BR-PDAC patients are often not immediately resectable, so NAT does not delay surgery but rather facilitates it. This is why the benefit appears more pronounced and consistent in this group.

Despite the growing body of evidence supporting NAT, concerns about its real-world feasibility remain significant. In multiple trials, a substantial proportion of patients randomized to NAT failed to reach surgical resection. This was most often due to disease progression during therapy, worsening performance status, or treatment-related toxicity, which resulted in treatment dropout or delays to surgery. In the NORPACT-1 trial, approximately 82% of patients with R-PDAC who received NAT underwent resection. The remaining patients were ineligible for surgery due to disease progression or deterioration of their clinical condition during treatment. In the meta-analysis conducted by Brown et al. [19] (2022), it was shown that the proportion of patients who did not undergo surgery after NAT was approximately 22.6% in the R-PDAC group and 39.4% in the BR-PDAC group. The main reason for abandoning surgery was disease progression, particularly the development of distant metastases or loss of local resectability. The resection rate was only 69% in the NEONAX study, as well as 68% in the PREOPANC study. These findings highlight that although NAT may improve margin-negative resection and lymph node clearance rates, its practical implementation is limited by attrition due to biological aggressiveness and patient fragility, factors not always captured in trial eligibility criteria. Therefore, treatment decisions should be individualized, weighing both oncologic benefits and patient-specific risks of delay or incomplete multimodal therapy. Careful patient selection and early evaluation of treatment response are essential to prevent overtreatment in patients unlikely to benefit from NAT.

## 7. Conclusions

**Figure 1:**
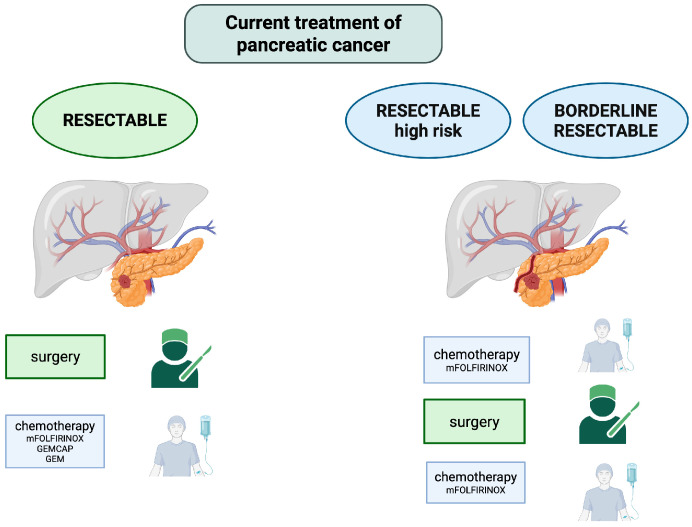
Current treatment of pancreatic cancer. Created with BioRender.com, accessed on 25 June 2025. Abbreviations: mFOLFIRINOX—modified FOLFIRINOX, GEMCAP—gemcitabine + capecitabine, and GEM—gemcitabine.

In summary, current guidelines increasingly emphasize the role of biological and radiological risk factors, such as elevated Ca 19-9 levels, radiological lymph node involvement, and tumor size ≥ T3, as indicators of more aggressive disease biology. For high-risk patients, NAT with FOLFIRINOX may be the best option, while for low-risk patients, upfront surgery remains a valid choice. In conclusion, current evidence supports the use of NAT as the standard of care for BR-PDAC. At the same time, the treatment of R-PDAC should be individualized based on risk stratification that incorporates both clinical and molecular features. The proposed treatment regimen is shown in Figure 1.

Evidence from the NORPACT-1 and PANACHE01-PRODIGE48 trials indicates that ctDNA positivity, both before and after treatment, correlates with a poor prognosis and a higher risk of recurrence. The integration of biomarkers such as ctDNA and CA 19-9, along with advances in immunotherapy, will be crucial in shaping the next generation of personalized treatment strategies. Ongoing trials, such as PANACHE01, NEOLAP, and ALLIANCE A021806, are incorporating biomarker designs to help personalize future treatments. Additionally, the future of treatment is likely to include immunotherapeutic and targeted approaches. Early-phase trials evaluating combinations of immune checkpoint inhibitors (nivolumab, urelumab) with tumor vaccines (GVAX) have demonstrated encouraging immunological responses; however, a small sample size and a lack of statistical power limit their findings. In addition, preliminary promising results from the new PAXG treatment regimen also represent a major shift in the approach to the landscape of NAT treatment.

**Figure 2:**
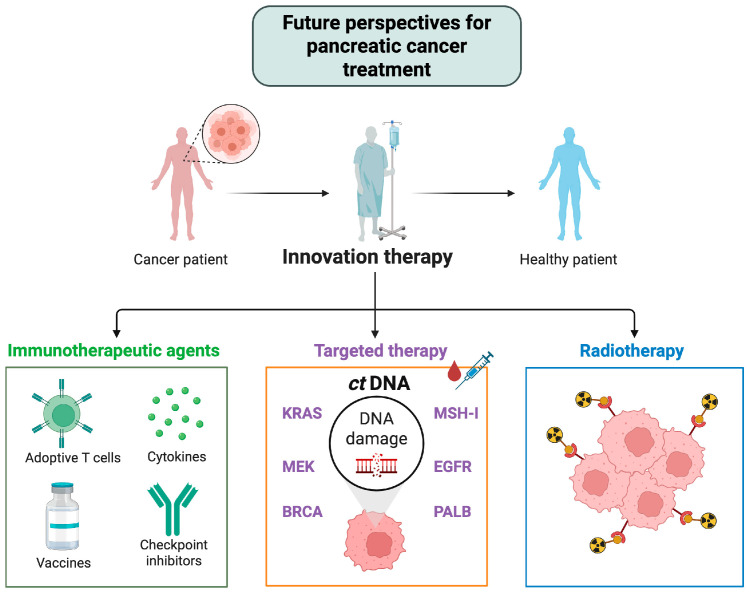
Future perspectives for pancreatic cancer treatment. Created with BioRender.com, accessed on 25 June 2025. Abbreviations: ctDNA—circulating tumor deoxyribonucleic acid, MEK—mitogen-activated protein kinase kinase, BRCA—breast cancer gene, KRAS—Kirsten rat sarcoma virus, PALB—partner and localizer of breast cancer gene, EGFR—epidermal growth factor receptor, and MSH-I—highly conserved MutS homologs.

These exploratory efforts highlight the need for larger, well-stratified trials to assess the effectiveness of new strategies in biomarker-selected patient populations. We hope that the future of patients with PC will resemble Figure 2.

## Figures and Tables

**Table 1:** NCCN criteria for PDAC staging.

Stage	Arterial Involvement	Venous Involvement
**Resectable**	No contact with the following: • CA, • SMA, • HA.	(≤180°) contact without contour irregularity
**Borderline resectable**	Head/uncinate process • Solid tumor contact with CHA without extension to CA or hepatic bifurcation, or • Abutment without extension to SMA or variant artery Body/tail • Abutment (≤180°) to the celiac axis or encasement without involving the aorta or gastroduodenal artery	>180° contact with contour irregularity or thrombosis, but resection and reconstruction are possible
**Unresectable**	Head/uncinate process • >180° contact with SMA or CA Body/tail • >180° contact with SMA or CA or • (≤180°) contact with CA plus aortic involvement	>180° contact or with contour irregularity or thrombosis, and resection and reconstruction are not possible

Abbreviations: CA—celiac artery, SMA—superior mesenteric artery, HA—hepatic artery, and CHA—common hepatic artery.

**Table 2:** Randomized controlled trials comparing adjuvant chemotherapy with observation for PDAC.

Trial/ Author	Phase	R/BR	Study Design	n	mOS	HR (95% CI)	*p*- Value	mDFS	HR (95% CI)	*p*- Value	R0
ESPAC-1 [14]	III	R	Observation CRT	69 63	17.9 15.9	1.28 (0.99–1.66)	0.05	15.2 10.7	1.32 (1.00–1.74)	0.04	NR
			Chemotherapy observation	75 72	20.1 15.5	0.71(055–0.92)	0.009	15.3 9.4	0.73 (0.55–0.96)	0.02	
CONKO-001 [8]	III	R	GEM	179	22.8	0.76 (0.61–0.95)	0.01	13.4	0.55 (0.44–0.69)	0.001	80%
			Observation	175	20.2			6.9			
JSAP-02 [15]	III	R	GEM	58	22.3	0.77 (0.51–1.14)	0.19	11.4	0.60 (0.40–0.89)	0.01	NR
			Observation	60	18.4			5.0			
ESPAC-3 [16]	III	R	5-FU	551	23.0	0.94 (0.81–1.08)	0.39	14.3	0.96 (0.86–1.07)	0.53	NR
			GEM	537	23.6			14.1			

Abbreviations: R—resectable, BR—borderline resectable, CRT—chemoradiotherapy, 5-FU—5-fluorouracil, GEM—gemcitabine, n—number of patients, vs.—versus, mOS—median overall survival, HR—hazard ratio, CI—confidence interval, mDFS—median disease-free survival, R0—resection margin 0 (no cancer cells seen microscopically at the primary tumor site), and NR—not reported.

**Table 3:** Randomized controlled trials comparing adjuvant chemotherapy for PDAC.

Trial/Author	Phase	Treatment	n	mOS (Months)	HR (95% CI)	*p*-Value	mDFS (Months)	HR (95% CI)	*p*-Value
PRODIGE24	III	mFOLFIRINOX	493	53.5	0.68 (0.54–0.85)	0.001	21.4	0.66 (0.54–0.82)	0.001
		GEM		35.5			12.8		
ESPAC-4	III	GEM	730	25.5	0.82 (0.68–0.98)	0.032	NR	NR	NR
		GEM-CAP		28.0			NR		

Abbreviations: mFOLFIRINOX—modified FOLFIRINOX, GEM—gemcitabine, CAP—capecitabine, n—number of patients, mOS—median overall survival, HR—hazard ratio, CI—confidence interval, mDFS—median disease-free survival, and NR—not reported.

**Table 4:** Selected randomized controlled trials comparing NAT with upfront surgery for R-PDAC and BR-PDAC.

Trial/ Author	Phase	R/ BR	Treatment Arm	n	mOS Months	HR (95% CI)	*p*- Value	mDFS Months	HR (95% CI)	*p*- Value	R0
Golcher et al. [23]	II	R	GEM/CIS+RTH/ surgery	73	17.4	NR	0.96	NR	NR	NR	52%
			Upfront surgery/GEM		14.4						48%
Casadei et al. [24]	II	R	GEM+RTH	38	NR	NR	0.174	NR	NR	NR	NR
			Upfront surgery/GEM		NR						
Jang et al. [25]	II/III	BR	GEM+RTH/surgery	110	21	1.495 (0.66–3.360	0.028	NR	NR	NR	52%
			Surgery/GEM+RTH		12						26%
Prep-02/JSAP-05 [26]	II	R/ RB	GEM+S-1/surgery	364	36.7	0.72 (0.55–0.94)	0.015	14.3	0.77 (0.61–0.98)	0.030	NR
			Surgery/S-1		26.6			11.3			
PREOPANC [27]	III	R/ BR	GEM+RTH/ Surgery/GEM	246	15.7	0.73 (0.58–0.96)	0.025	8.1	0.70 (0.53–0.92)	0.009	71%
			Surgery/GEM		14.3			7.7			40%
PREOPANC-2 [28,29]	III	R/ BR	FOLFIRINOX/ SURGERY	368	21.9	0.87 (0.68–1.12)	0.28	NR	NR	NR	77%
			GEM+RTH/ surgery		21.3						75%
NORPACT-1 [30]	II	R	FOLFIRINOX/ surgery/adjuvant chemotherapy	140	25.1	1.52 (1.00–2.33)	0.05	NR	NR	NR	82%
			Surgery/FOLFIRINOX		38.5						89%
SWOG S1505 [31]	II	R	FOLFIRINOX/surgery/FOLFIRINOX	102	22.4	0.97 (0.76–1.24)	0.82	10.9	0.87 (0.66–1.15)	0.87	85%
			NP/surgery/NP		23.6			14.2			85%
NEONAX [32]	II	R	NP/surgery	127	25.2	(19.0–29.7)	0.028	11.5	(8.8–14.5)	NR	88%
			Surgery/NP		16.7	(11.6–22.2)		5.9	(3.6–11.5)		67%
ALLIANCE A021501 [34]	II	BR	FOLFIRINOX/ surgery	126	29.8	1.88 (1.16–3.04)	0.009	15.0	1.28 (0.88–1.87)	NR	57%
			FOLFIRINOX+SBRT/surgery		17.1			11.5			33%
CASSANDRA [35]	III	R/ BR	PAXG/surgery mFOLFIRINOX	261	37.3	0.7 (0.47–1.04)	0.07	17.3	0.64 (0.46–0.89)	0.008	74%
					26.0			10.4			51%

Abbreviations: R—resectable, BR—borderline resectable, RTH—radiotherapy, GEM—gemcitabine, CIS—cisplatin, S-1—oral fluoropyrimidine-based chemotherapy, NP—nab-paclitaxel, PAXG—(cisplatin, nab-paclitaxel, capecitabine, gemcitabine), n—number of patients, vs. versus, mOS—median overall survival, HR—hazard ratio, CI—confidence interval, mDFS—median disease-free survival, R0—resection margin 0 (no cancer cells seen microscopically at the primary tumor site), and NR—not reported.

**Table 5:** Meta-analyses of clinical trials.

Author	Trials (n)	Number of Patients	R/ BR	Treatment Arm	mOS	HR (95% CI)	*p*- Value	mDFS	HR (95% CI)	*p*- Value	R0	Conclusion
Versteijne et al. [37]	38	3484	R/ RB	NAT	18.8	0.73	NR	NR	NR	NR	87%	NAT may improve OS and R0 resection in BR-PDAC
				Upfront surgery	14.8						67%	
Aliseda et al. [38]	5	625	R	NAT	25.9	0.88 (0.72–1.08)	0.223	NR	NR	NR	NR	No significant OS benefit from NAT in R-PDAC
				Upfront surgery	23.8							
Uson Junior et al. [39]	6	805	R	NAT	NR	0.76 (0.52–1.11)	NR	NR	0.71	NR	↑20% with NAT	No OS/DFS improvement; improved R0 rate
				Upfront surgery								
Dickerson et al. [40]	9	1194	R/ BR	NAT	NR	0.73 (0.55–0.98)	NR	NR	NR	NR	NR	No significant OS benefit in R-PDAC
				Upfront surgery								
Chan et al. [41]	8	982	R	NAT	NR	0.81 (0.65–1.01)	0.06	12.7	0.66 (0.47–0.92)	0.01	72%	DFS, R0, and N0 improved; no significant OS difference.
				Upfront surgery				6.3			60%	
Cloyd et al. [42]	6	850	R/ BR	NAT	25.4	0.73 (0.61–0.86)	0.001	NR	NR	NR	60%	OS, R0 benefit in R-PDAC and BR-PDAC
				Upfront surgery	19.4						40%	

Abbreviations: R—resectable, BR—borderline resectable, NAT—neoadjuvant therapy, n—number of patients, mOS—median overall survival, HR—hazard ratio, CI—confidence interval, mDFS—median disease-free survival, R0—resection margin 0 (no cancer cells seen microscopically at the primary tumor site), and NR—not reported.

**Table 6:** Ongoing randomized controlled trials comparing neoadjuvant chemotherapy with upfront surgery for R-PDAC and BR-PDAC.

Trial	Phase	R/BR	Treatment	n	Primary Outcome	Secondary Outcomes
NCT04927780	III	R	FOLFIRINOX/surgery /FOLFIRINOX	378	OS	PFS, number of cycles received, dose intensity, resection rate, quality of life, AE, and surgical complications.
			Surgery/FOLFIRINOX			
NCT04340141	III	R	FOLFIRINOX/surgery /FOLFIRINOX	352	OS	DFS, resection rate, AE, and quality of life.
			Surgery/FOLFIRINOX			
NCT02172976	II/III	R	FOLFIRINOX/surgery/ FOLFIRINOX	40	OS	PFS, peri-operative morbidity and mortality, R0 RR, tolerability, and feasibility of neoadjuvant FOLFIRINOX
NCT01314027	III	R	GEM/oxaliplatin	38	PFS	-
			Upfront surgery			
NCT02676349	II	BR	mFOLFIRINOX+ CAP-based CRT/surgery	90	R0 RR	-
			mFOLFIRINOX/surgery			
NCT02717091	II	BR	FOLFIRINOX	50	R0 RR	-
			NP/GEM			

Abbreviations: R—resectable, BR—borderline resectable, n—number of patients, OS—overall survival, DFS—disease-free survival, PFS—progression-free survival, CAP—capecitabine, NP—nab-paclitaxel, GEM—gemcitabine, AE—adverse events, RR—resection rate, R0—resection margin 0 (no cancer cells seen microscopically at the primary tumor site).

**Table 7:** Clinical trials with immunotherapy.

Author	Phase	n	R/BR/LA	Treatment Arm	Outcomes	
Du et al. [51]	II	29	RB/ LA	tislelizumab +GEM/NP +SBRT-SIB	ORR: 60% R0: 90% 12-Month OS Rate: 72% 12-Month PFS Rate: 64%	
Heumann et al. [52]	Ib/II	40	R	Arm A: GVAX+cyclofosphamide	mOS: Arm A: 23.59 m Arm B: 27.01 m Arm C: 35.55 m	mDFS: Arm A: 13.90 m Arm B: 14.98 m Arm C: 33.51 m
				Arm B: GVAX+cyclofosphamide + nivolumab		
				Arm C: GVAX+cyclofosphamide + nivolumab + urelumab		
Byrne et al. [53]	I/II	16	R	Arm A: selicrelumab	mOS: 95% CI (18.0–28.8 m) Arm A: 23.4 m Arm B: not reached	mDFS: 95% CI (0.4–19.2 m) Arm A: 9.8 m Arm B: not reached
				Arm B: selicrelumab+GEM+NP		

Abbreviations: R—resectable, BR—borderline resectable, LA—locally advanced, GEM—gemcitabine, NP—nab-paclitaxel, GVAX—GM-CSF-secreting allogeneic pancreatic tumor cells, n—number of patients, mOS—median overall survival, PFS—progression-free survival, mDFS—median disease-free survival, and CI—confidence interval.

**Table 8:** Ongoing clinical trials with immunotherapy for early PDAC.

Trial	Phase	R/RB/LA	Treatment	Primary Endpoints
NCT03983057	II	BR/LA	mFOLFIRINOX/surgery mFOLFIRINOX + PD-1 antibody/surgery	PFS, TTP
NCT05132504	II	R	mFOLFIRINOX + Pembrolizumab/surgery	Safety
			upfront surgery/mFOLFIRINOX + Pembrolizumab	
NCT06094140	II	R/BR	mFOLFIRINOX+Durwalumab/surgery	Safety
NCT06060405	II	R	Durwalumab+Oleclumab/surgery	Change in CD8+ T cells within tumor,
NCT00727441	II	R/BR	GVAX GVAX, cyclophosphamid i.v. GVAX, cyclophosphamid p.o	Safety, immune response
NCT03727880	II	R	Chth NAT + pembrolizumab + defactinib	Change in CD8+ T cells within tumor, pCR
			Chth NAT + pembrolizumab i.v	
NCT03979066	II	R	Atezolizumab + PEGPH20	Change in CD8+ T cells within tumor
			Atezolizumab i.v.	
NCT03970252	II	BR	FOLFIRINOX+Nivolumab	Evaluation of development of clinically relevant pancreatic fistula in the post-operative period Evaluation pCR
NCT02305186	Ib/II	R/BR	CRT (capecitabine) + Pembrolizumab	Number of Tumor Infiltrating Lymphocytes (TILs) per high powered field (hpf) in pancreatic tissue (resected tissue) Safety: Incidence of Dose-Limiting Toxicities (DLTs)
			CRT	
NCT04940286	II	R/BR	GEM+NP+Durvalumab+Oleclumab/Surgery/adjuvant	Major pathological response rate (=<5% viable tumor cells) Incidence of adverse events

Abbreviations: R—resectable, BR—borderline resectable, LA—locally advanced, GEM—gemcitabine, NP—nab-paclitaxel, GVAX—GM-CSF-secreting allogeneic pancreatic tumor cells, n—number of patients, PFS—progression-free survival.

**Table 9:** Ongoing clinical trials with target therapy for early PDAC.

Trial	Phase	R/BR/LA	Indication Group	Treatment	Primary Endpoints
NCT04858334	II	R/BR	BRACA1/2 or PALB2, post platinum-based NAT chemotherapy, and resection	olaparib	RFS
NCT04005690	I	R/BR/LA	MEK inhibitor PARP inhibitor PLK1 inhibitor WEE1/Cell-Cycle Checkpoint Inhibition Anti-CTLA-4 antibody	cobimetinib olaparib/saruparib onvansertib azenosertib tremelimumab	Proportion of pharmacodynamic feasibility
NCT05546411	I/II	R/BR/LA	TGF-beta inhibitor	NIS793	MPR
NTC04117087	I	R/BR/LA/metastatic	MMR-p	KRAS peptide vaccine +nivolumab+ipilimumab	AE

Abbreviations: R—resectable, BR—borderline resectable, LA—locally advanced, RFS—relapse-free survival, MPR—major pathological response, MMR-p—mismatch repair protein, and AE—adverse events.

## Abbreviations

The following abbreviations are used in this manuscript:

PDAC	pancreatic ductal adenocarcinoma cancer
NAT	neoadjuvant treatment
PC	pancreatic cancer
R-PDAC	resectable pancreatic ductal adenocarcinoma
BR-PDAC	borderline resectable pancreatic ductal adenocarcinoma
R0	resection margin 0 (no cancer cells seen microscopically at the primary tumor site)
LA-PDAC	locally advanced pancreatic ductal adenocarcinoma
RCTs	randomized controlled trials
PRISMA	Preferred Reporting Items for Systematic Reviews and Meta-Analyses
5-FU+LV	5 fluorouracil plus leucovorin
GEM	gemcitabine
RES	recurrence-free survival
CRT	chemoradiotherapy
Mos	median overall survival
HR	hazard ratio
CI	confidence interval
mDFS	median disease-free survival
NR	not reported
ITT	intention-to-treat
MDCT	multidetector computed tomography
SMA	superior mesenteric artery
MRI	magnetic resonance imaging
CT	computed tomography
NCCN	National Comprehensive Cancer Network
CHA	common hepatic artery
SMV	superior mesenteric vein (SMV)
PV	portal vein
NICE	National Institute for Health and Care Excellence
ESMO	European Society for Medical Oncology
ICIs	immune checkpoint inhibitors
SBRT	SIB simultaneous integrated boost
Ct-DNA	circulating tumor DNA
maxVAF	maximum somatic variant allele frequency
GVAX	tumor vaccines
EGFR	epidermal growth factor receptor
IGF-1	insulin growth factor
VEGF	vascular endothelial growth factor
